# How Diverse Detrital Environments Influence Nutrient Stoichiometry between Males and Females of the Co-Occurring Container Mosquitoes *Aedes albopictus*, *Ae*. *aegypti*, and *Culex quinquefasciatus*

**DOI:** 10.1371/journal.pone.0133734

**Published:** 2015-08-05

**Authors:** Donald A. Yee, Michael G. Kaufman, Nnaemeka F. Ezeakacha

**Affiliations:** 1 Department of Biological Sciences, University of Southern Mississippi, Hattiesburg, Mississippi, United States of America; 2 Department of Entomology, Michigan State University, East Lansing, Michigan, United States of America; New Mexico State University, UNITED STATES

## Abstract

Allocation patterns of carbon and nitrogen in animals are influenced by food quality and quantity, as well as by inherent metabolic and physiological constraints within organisms. Whole body stoichiometry also may vary between the sexes who differ in development rates and reproductive allocation patterns. In aquatic containers, such as tree holes and tires, detrital inputs, which vary in amounts of carbon and nitrogen, form the basis of the mosquito-dominated food web. Differences in development times and mass between male and female mosquitoes may be the result of different reproductive constraints, which could also influence patterns of nutrient allocation. We examined development time, survival, and adult mass for males and females of three co-occurring species, *Aedes albopictus*, *Ae*. *aegypti*, and *Culex quinquefasciatus*, across environments with different ratios of animal and leaf detritus. We quantified the contribution of detritus to biomass using stable isotope analysis and measured tissue carbon and nitrogen concentrations among species and between the sexes. Development times were shorter and adults were heavier for *Aedes* in animal versus leaf-only environments, whereas *Culex* development times were invariant across detritus types. *Aedes* displayed similar survival across detritus types whereas *C*. *quinquefasciatus* showed decreased survival with increasing leaf detritus. All species had lower values of ^15^N and ^13^C in leaf-only detritus compared to animal, however, *Aedes* generally had lower tissue nitrogen compared to *C*. *quinquefasciatus*. There were no differences in the C:N ratio between male and female *Aedes*, however, *Aedes* were different than *C*. *quinquefasciatus* adults, with male *C*. *quinquefasciatus* significantly higher than females. *Culex quinquefasciatus* was homeostatic across detrital environments. These results allow us to hypothesize an underlying stoichiometric explanation for the variation in performance of different container species under similar detrital environments, and if supported may assist in explaining the production of vector populations in nature.

## Introduction

Food quality has clear effects on performance of consumers, and diet has the potential to be an important factor for the evolution and diversification of life forms. Food itself can be considered in light of its elemental composition and its contribution to consumer nutrient composition, thus helping to achieve a deeper understanding of consumer properties at all levels of organization [1]. In both aquatic and terrestrial systems, primary productivity provides the bulk of the resources available to consumers. However, detrital pathways often dominate nutrient flow in any given system and allocthonous detritus serves as the nutritional foundation in many systems [2]. Aquatic containers, including both natural (tree holes, pitcher plants) and artificial (discarded automobile tires, flower vases), rely on these allochthonous inputs of detritus (e.g., senescent leaves, flowers, invertebrate carcasses) that serve as the main nutritional base for developing insect larvae [3–5]. Detrital inputs vary in their composition and rates of decomposition, and serve as a resource for the growth of fungi, bacteria, and protozoans, all of which are key food resources for invertebrates [5–7]. These detritus types can vary considerably in their nutrient content [8, 9], and therefore produce variable effects on the performance of consumers [9, 10]. What remains unknown in these systems is how nutrient signatures of consumers vary with detritus type, or how much variation in those signatures exist among species.

Aquatic containers are used by several dozen species of medically important mosquitoes [11–12]. Three of the most important cohabiting container species are *Aedes aegypti* (yellow fever mosquito), *A*. *albopictus* (Asian tiger mosquito), and *Culex quinquefasciatus* (southern house mosquito) [13, 14]. *Aedes aegypti* is an established species from Africa, introduced to the United States during the slave trade [15] and was historically the dominant container species in the eastern United States. It is closely associated with human habitation and has become the major urban vector of several human arboviruses worldwide [13]. In the United States, the abundance and geographic range of *A*. *aegypti* has substantially decreased, sometimes to the point of local extinction, since the invasion of *A*. *albopictus* [16, 17]. *Aedes albopictus* is an invasive species from southeast Asia, and was introduced into the United States in the mid-1980s through an international shipment of tires [18]; it is a competent vector of a suite of arboviruses [19–23]. Although *A*. *albopictus* has replaced *A*. *aegypti* as the dominant container species in the eastern United States, both species co-occur in a few urban populations in the southeast, especially around the city of New Orleans, Louisiana and southern Florida [16, 17]. *Aedes albopictus* is the second most common container species in the U.S. and also the most abundant species in tires in the southeast [12, 24]. Multiple laboratory and field trials have established *A*. *albopictus* as a superior resource competitor to several native and established species including *A*. *aegypti*, *A*. *triseriatus*, *Culex pipiens*, and *Culex quinquefasciatus* [17, 25–32]. However, this competitive superiority is context-dependent, and outcomes can change depending on detritus type and amount [32–34] and other factors related to the environment [31, 35]. In discarded vehicle tires in the eastern United States, *A*. *albopictus* in addition to other *Aedes*, often frequently co-occurs with *Culex* sp., notably *Cx quinquefasciatus* [36, 37]. *Culex quinquefasciatus* is predominantly an urban species found in subtropical and tropical regions of the world [38, 39] and is one of the most widely distributed members of the *Culex pipiens* species complex [39]. *Culex quinquefasciatus* is a medically important vector of some important arboviruses [40]. Tests of competitive interactions with *A*. *albopictus* have shown that some *Culex*, including *C*. *coronator* [41] and *C*. *quinquefasciatus* [32] to be the inferior competitor under some detrital environments.

It is assumed that species respond to certain environmental conditions in a predictable way, however differences between males and females of the same species in some life-history traits can occur. For instance, in many arthropods, protandry, or the “emergence of males prior to females into seasonally breeding populations” [42], may produce differences in life history traits such as mass, in the two sexes. In the mosquito *Aedes sierrensis* males develop faster but at the cost of smaller size, to gain access to virgin females, but females lengthen develop times to maximize mass, the latter attribute being positively related to lifetime fecundity [42, 43]. Yee and co-authors [43] documented development time differences in male and female *A*. *albopictus* under different simulated seasonal photoperiods. In addition, they showed that female mass and development times were more plastic across photoperiods, whereas male development times were less variable across photoperiods [43]. Because of protandry, we might predict that males and females differ in their assimilation of nutrients sequestered from microorganisms or from detritus. Specifically, males, having been selected for quick development time at the cost of size, may reach their minimum nutrient threshold sooner, irrespective of detritus type and its nutrient availability. In fact, male *A*. *albopictus* differed in development time but not mass among different rearing photoperiods, whereas females varied in both attributes [43]. This suggest that once males reach a critical minimum size they complete development and emerge as adults regardless of the quality of the nutrient environment. Conversely, females, selected for maximum size with increased development time, may have higher nutrient thresholds than males or may stockpile some elements to allocate to reproductive tissues or products. Several studies have documented sex-specific trade-offs in development time and mass in *A*. *albopictus* under a variety of biotic [44] and abiotic conditions [31, 45]. However, it is still poorly understood if sex-specific mass development trade-offs are affected by multiple detritus types, and if such trade-offs produce differences between males and females in their nutrient stoichiometry.

Stable isotope analysis is a recent but potentially important tool to understand the biology and ecology of animals, including arthropods [46]. This analysis relies on quantifying isotopes of several elements, including carbon (^13^C/^12^C, expressed as δ^13^C) and nitrogen (^15^N/^14^N, expressed as δ^15^N) assimilated by consumers. Carbon can be used to infer the energy source for higher consumers because δ^13^C is often conserved from food source to consumer, but can still vary among food sources [47, 48]. Nitrogen (^15^N) for consumers is often enriched 3–4‰ relative to their food, making nitrogen stable isotope ratios useful in determining consumer trophic position. Several recent studies have used stable isotope or nutrient analysis to understand the ecology of mosquitoes [8, 9, 49, 50]. These studies have collectively determined that variation exists among different mosquito species, reflected in patterns of nutrients in the adult body resulting from natural or artificial diets. However, none of these studies have simultaneously assessed differences between males and females for different container genera.

We examined the performance of *A*. *aegypti*, *A*. *albopictus*, and *C*. *quinquefasciatus* grown on two common detritus types found in containers, invertebrate carcasses and senescent deciduous leaves, at different ratios. We specifically hypothesized that (i) variation in animal and leaf detritus will alter the performance (survival, development times, mass) of these mosquito species, and (ii) there will be different stoichiometric patterns in males and females due to sex-specific trade-offs. We predicted that as males often develop faster and are smaller than females they would have nutrient signatures different than females. We used stable isotope analysis of ^13^C and ^15^N values as well as tissue carbon and nitrogen in adults to quantify nutrient assimilation from detrital source. This allows us to link mosquitoes to their food source [8, 9] and can help to elucidate mechanisms of differential growth performance of males and females on different detritus ratios. We found support for our first hypothesis in that mosquitoes had higher survival, shorter development times, and were larger in animal versus leaf detritus environments. Moreover, although there were no differences in stoichiometry between the sexes for either *Aedes* species, *Culex* males did show a higher C:N ratio regardless of the detrital environment, at least partially supporting our second hypothesis.

## Materials and Methods

### Study design

Second generation (F_2_) eggs of *Aedes albopictus* and *A*. *aegypti* were obtained from laboratory colonies at Vero Beach, Florida, USA, whereas *Culex quinquefasciatus* egg rafts were from a laboratory colony established from Florida since 1985. All eggs were hatched in a solution of 0.33 g of Nutrient Broth (Difco, BD, Sparks, MD, USA) and 750 ml of reverse-osmosis (RO) water after which all first-instar larvae were rinsed after hatching to remove nutrient broth. Twenty individuals of each species were placed separately into 250 ml tripour beakers containing animal detritus (freeze-dried crickets (*Gryllodes sigillatus)*, Fluker Farms, Port Allen, LA, USA) and leaf detritus (senescent red maple (*Acer rubrum*) collected at the Lake Thoreau Center, Hattiesburg, MS, USA, 31°19’37.63”N, 89°17’25.22”W). Lake Thoreau is managed by the University of Southern Mississippi and as faculty and students we are allowed open access for scientific work. The field collection of leaves for experiments did not involve endangered or protected species. Detritus types were expressed as four different ratios in relative terms: 2:0, 1:1, 2:10 and 0:10 animal:leaf (1 unit of detritus equals 0.10 g). This produced 0.005 g per unit of detritus per larva (0.10g/20 larvae), an amount equal to a previous study [9], however the total amount of detritus varied among different detritus ratios. Each treatment level was replicated six times for a total of 24 beakers per species. Twenty-four hours prior to larval addition, each beaker was filled with 199 ml of reverse osmosis (RO) water and detritus and 1 ml of homogenized tire inoculum obtained from several field tires to allow for microorganism growth. Water levels were maintained at 200 ml through regular additions of RO water. We placed all beakers into each of three trays in an environmental chamber (Percival Scientific, Inc., Perry, IA, USA) set to 20°C on a 12 h: 12 h light:dark cycle [9]. Trays were rotated daily in a clockwise manner within the environmental chamber. The experiment ran for 40 days.

When present, pupae were removed and placed individually in shell vials until adult eclosion. Adults were identified to sex and species, freeze-killed and dried for 48 hrs at 50°C after which time their dry weights were measured using a XP2U ultra-microbalance (Mettler Toledo Inc., Columbus, Ohio). For each detritus treatment level, we measured male and female development times (days from egg hatch to adult eclosion), dry mass (mg), and survivorship (percentage of initial larvae surviving to adulthood).

For stable isotope analysis, mosquitoes and detritus were prepared by drying in an oven for ≥ 48 hrs at 50°C. For each detritus ratio within each species we measured out 1.25 mg each of dry male and female mosquito tissue from each of three replicate samples for analysis. This 1.25 mg represented multiple adult mosquitoes, and when we exceeded this amount different body parts (legs, wings) from different individuals were removed to meet our target mass. Based on different data, a leg typically contributed <3% to total body mass, and thus the small contribution that a leg or other body part would have to total values, and the fact that all replicates were handled in the same way and yielded low variation among replicates, suggests that our preparation technique would have little impact on the final values. For the detritus, we measured out four samples each of 1.25 mg dry maple leaf and 4.00 mg cricket. Mosquito tissue and detritus samples were analyzed for stable isotope (*δ* ^15^N and *δ* ^13^C) and total nutrient (C and N, in μg) analysis by the University of California Davis Stable Isotope Facility. Analysis was done on a PDZ Europa ANCA-GSL elemental analyzer interfaced to a PDZ Europa 20–20 isotope ratio mass spectrometer (Sercon Ltd, Crewe, U.K.).

### Statistical analysis

After (x+1)^2^ transformation of mosquito adult mass and development times to meet assumptions of normality and homogeneity of variances, we used multivariate analysis of variance (MANOVA) to test the null hypothesis that adult mass and development times for male and female mosquitoes were equal among detritus ratios. This was done separately for each species. We used standardized canonical coefficients (SCC) to indicate the important variables accounting for observed multivariate effects [51].

Analysis of variance (ANOVA) was used to test the null hypothesis of equal survival for each species among detritus ratios, after an arcsine square root data transformation to meet assumptions of normality and homogeneous variances. Differences in survival among treatments or species were identified using the Tukey-Kramer HSD post-hoc test for multiple comparisons.

To test the null hypothesis that the stable isotope ratios (*δ* ^15^N and *δ* ^13^C) and nutrient signatures (C and N) were the same for adult mosquitoes among the detritus ratios for each species, MANOVA was used after log transformation of data to meet assumptions. Because of lower survival in the 0:10 ratio, only one replicate of *C*. *quinquefasciatus* was available for analysis. In addition, due to mishandling of samples, two replicates in the 1:1 *A*. *aegypti* treatment level were not used in any nutrient analysis. We analyzed the carbon to nitrogen ratio (C:N) based on mass among species, sex, and across detritus ratios using ANOVA, and identified significant differences among means using Tukey-Kramer tests. Values of the nutrient ratio were log-transformed to meet assumptions.

## Results

There were significant effects of detritus ratios on the development time and mass for male and female *A*. *aegypti* (Pillai’s Trace_12, 57_ = 1.632, *P* < 0.001), *A*. *albopictus* (Pillai’s Trace_12, 57_ = 1.516, *P* < 0.001) and *C*. *quinquefasciatus* (Pillai’s Trace_12, 57_ = 1.261, *P* < 0.001). Development time (male SCC = 2.545, female = 3.926) contributed more to the significant MANOVA effects in *A*. *aegypti*, compared to adult mass (male SCC = -1.508, female = -0.031). For *A*. *albopictus*, male development time (SCC = 4.592) and mass (SCC = -2.174) accounted for the significant effects, compared to female development time (SCC = -1.038) and mass (SCC = -1.931). For *C*. *quinquefasciatus* however, adult mass (male SCC = 4.322, female = 3.045) contributed more to the significant MANOVA effects, compared to development time (male SCC = -1.963, female = -0.977).

**Fig 1:**
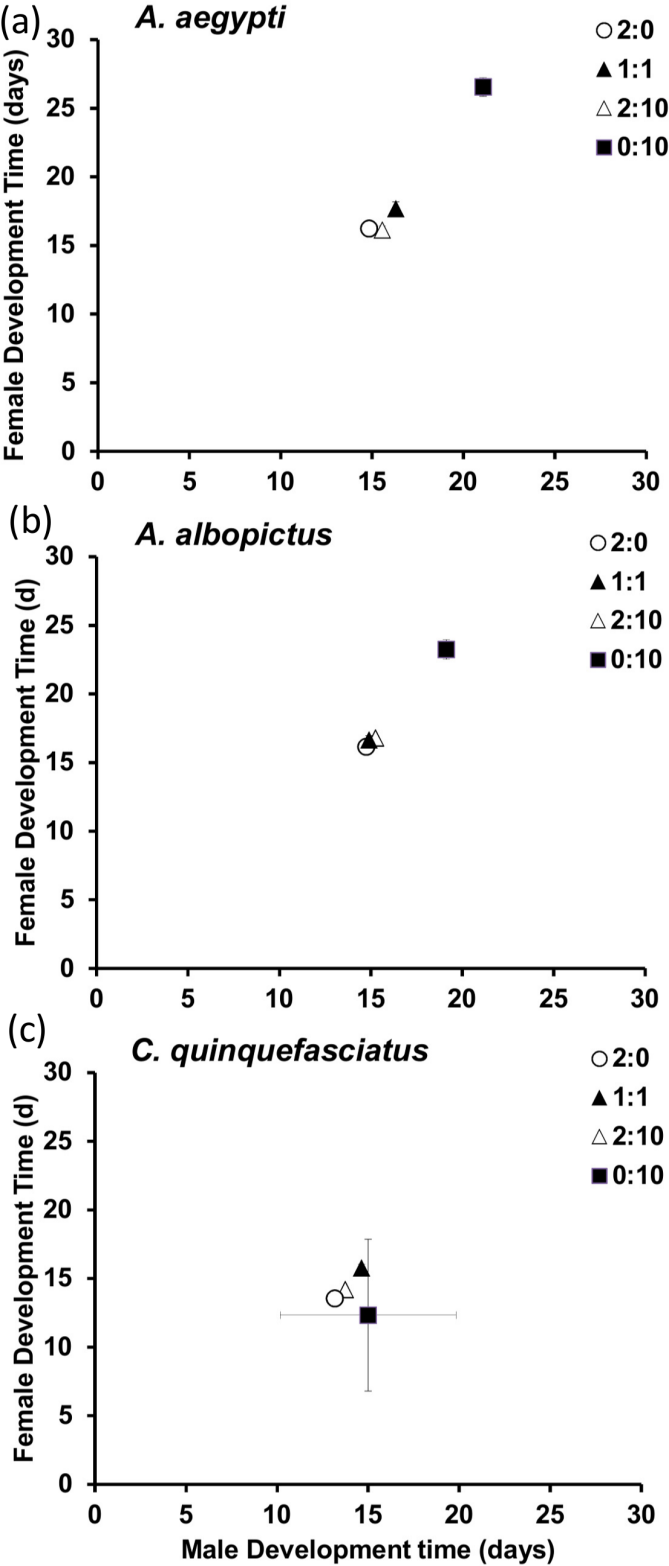
Development times (mean ± SE) for male and female (a) *Aedes aegypti*, (b) *Aedes albopictus* and (c) *Culex quinquefasciatus*, across detritus ratios (animal:leaf). Detritus ratios are expressed in units, where one unit = 0.10 g.

Based on post hoc tests, development times of males and females were different across detritus ratios. This difference was significant in *A*. *aegypti* and *A*. *albopictus*, with adult males and females from leaf-only containers (0:10) taking the longest time to develop compared to other detritus treatment levels (Fig 1A and 1B). However, *C*. *quinquefasciatus* development times for males and females did not differ across detritus combinations (Fig 1C).

**Fig 2:**
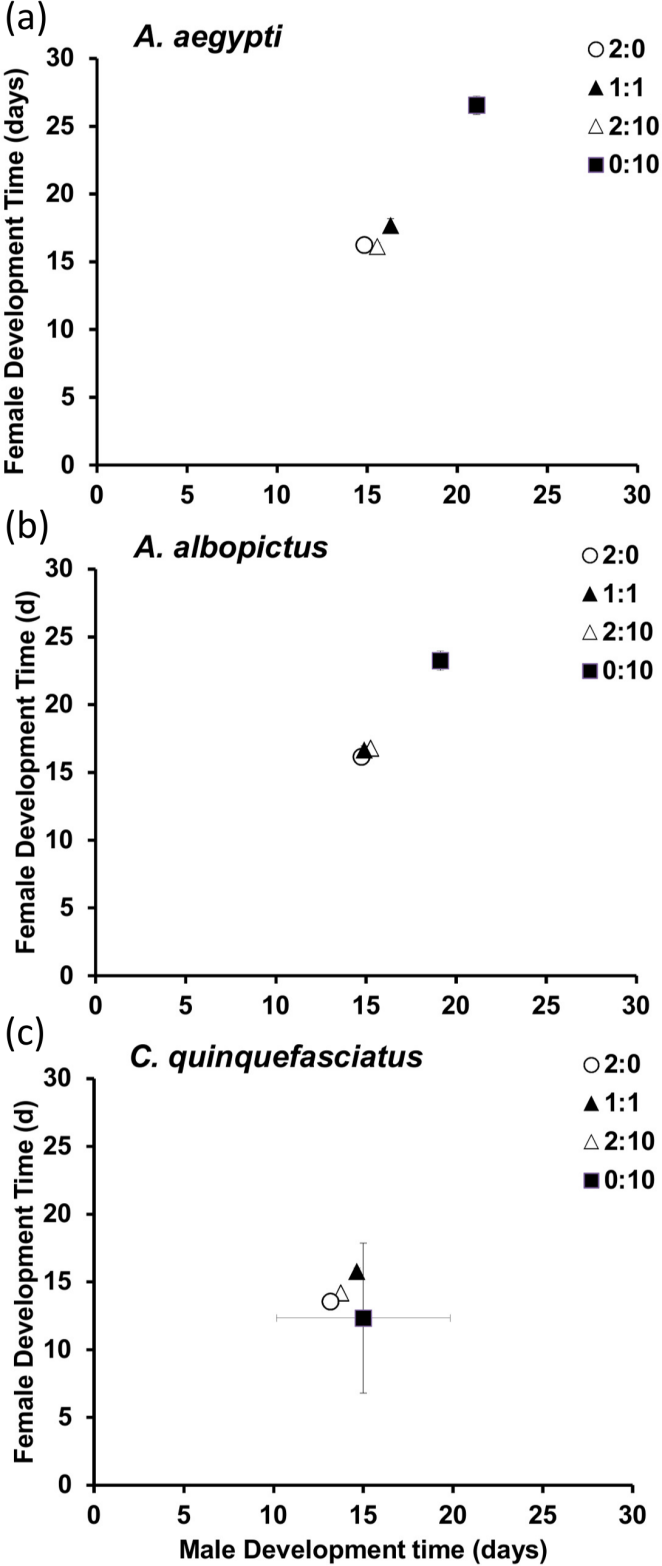
Mass (mean ± SE) for male and female (a) *Aedes aegypti*, (b) *Aedes albopictus* and (c) *Culex quinquefasciatus*, across detritus ratios (animal:leaf). Detritus ratios are expressed in units, where one unit = 0.10 g.

Male and female mass also differed across detritus ratios, with mosquitoes reared with some animal detritus having higher mass than leaf-only containers (Fig 2). Specifically, in the leaf-only containers (0:10), males and females had the lowest mass for all three species (Fig 2). However, adult mass was significantly higher in males and females from containers with combinations of animal and leaf detritus (i.e., 1:1, 2:10) (Fig 2). Within species, the response of adult mass to detritus ratios differed significantly between sexes. In *A*. *aegypti*, female mass was highest in the high animal-leaf ratio (2:10) whereas male mass was highest in the animal-only ratio (2:0) (Fig 2A). Conversely, female mass was highest in the animal-only ratio (2:0), for *A*. *albopictus* and *C*. *quinquefasciatus* (Fig 2B and 2C). Male mass was also highest in the animal-only ratio (2:0) for *A*. *albopictus*, but in *C*. *quinquefasciatus* the highest male mass was from the high animal-leaf ratio (2:10).

**Fig 3:**
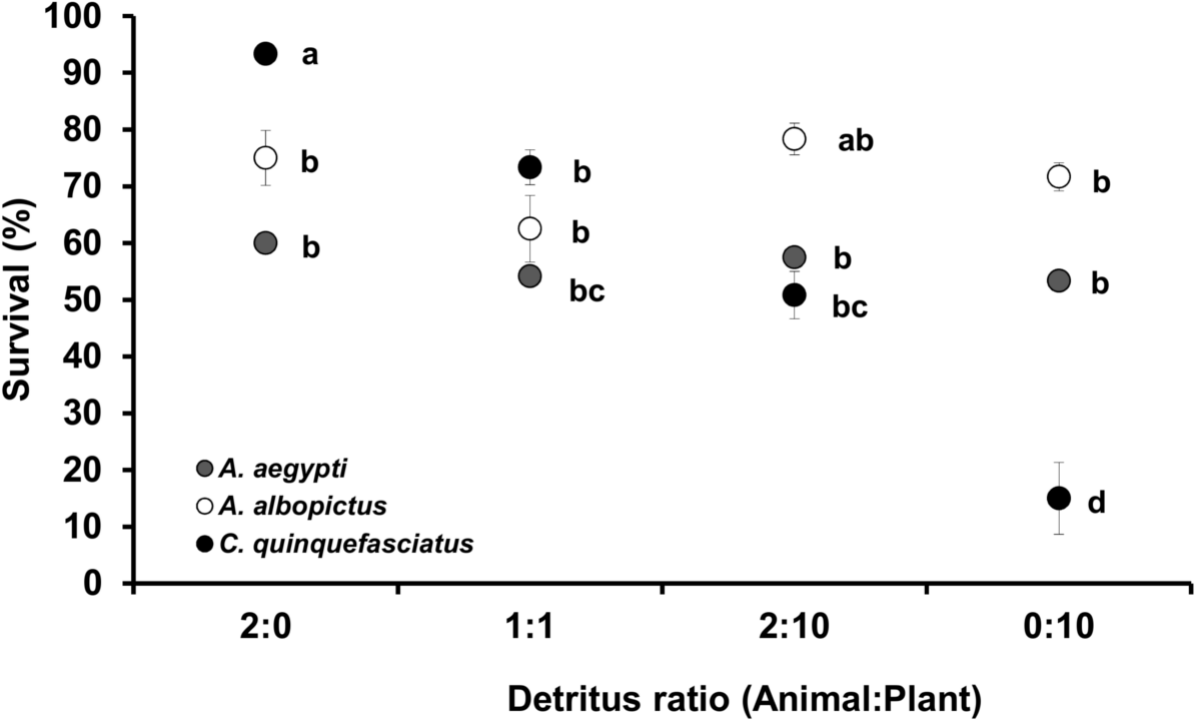
Survivorship (mean ± SE of percentage surviving) of *Aedes aegypti*, *Aedes albopictus* and *Culex quinquefasciatus* across animal and leaf detritus ratios. Letters represent Tukey-post Hoc test results. Means sharing same letters are not significantly different. Detritus ratios are expressed in units, where one unit = 0.10 g.

Survival differed significantly among species (F_2, 71_ = 11.41, *P* < 0.001), detritus ratio (F_3, 71_ = 23.69, P <0.001), and species-detritus ratio interaction (F_6, 71_ = 19.67, *P* < 0.001). Among species, *C*. *quinquefasciatus* had the highest survival in the animal-only treatment level, but was significantly lower in the leaf-only level, with mixtures producing intermediate survival compared to *Aedes*. Within species, survival of *C*. *quinquefasciatus* generally declined with decreasing animal detritus across ratios, with the highest survival in animal-only (2:0), the lowest survival in the leaf-only (0:10), with others intermediate (Fig 3). For *Aedes*, survival was similar across detritus ratios, although *A*. *albopictus* generally had higher survival than *A*. *aegypti* especially in high animal and leaf ratio (2:10) (Fig 3).

**Fig 4:**
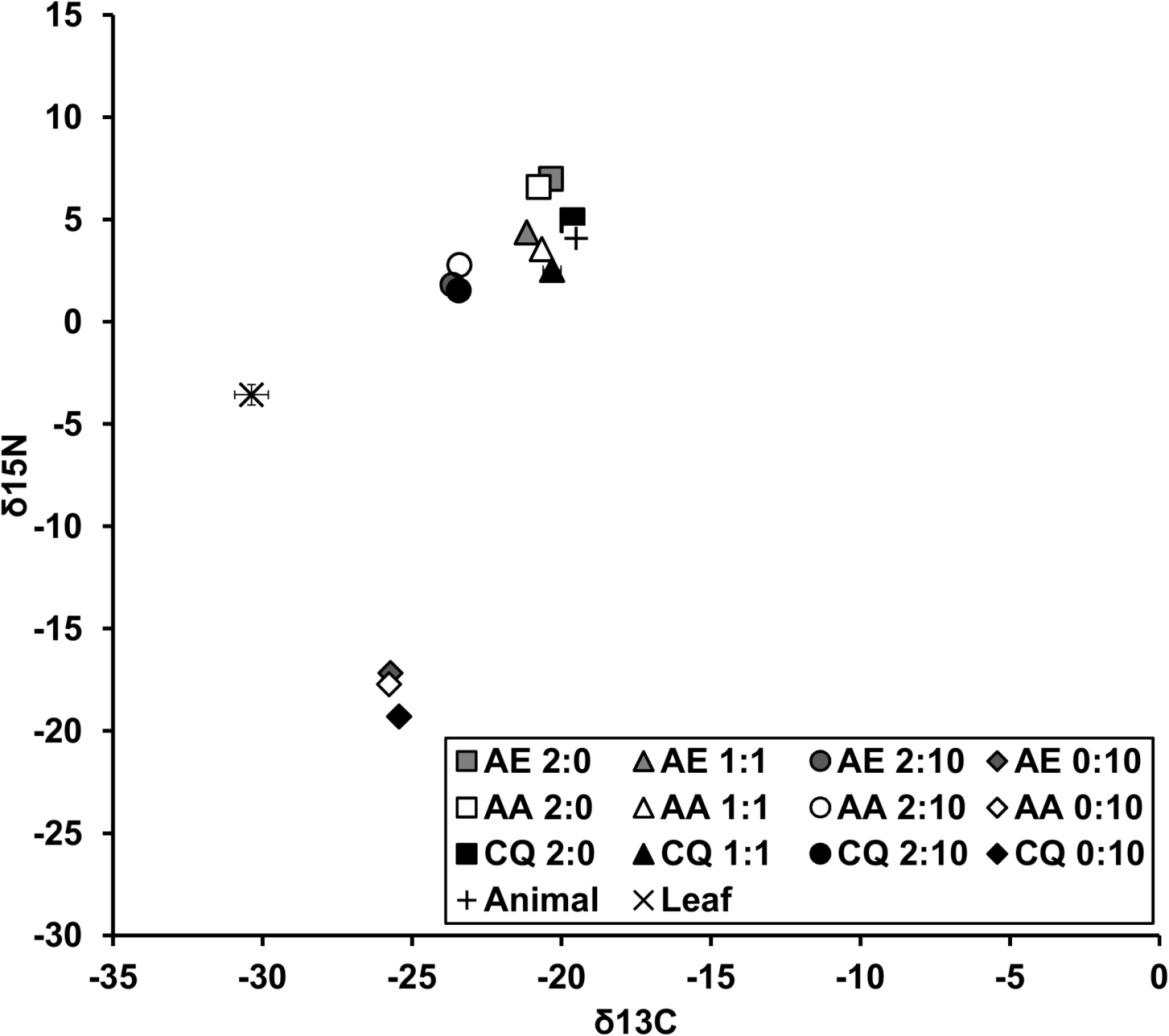
Bi-plot of stable isotope composition of detritus and adult *Aedes aegypti* (AE), *A*. *albopictus* (AA), and *Culex quinquefasciatus* (CX) across detritus ratios (animal:plant). Values are means ± SE from three replicates (except AE 1:1 and CX 0:10 which each had only one sample). Detritus ratios are expressed in units, where one unit = 0.10 g.

Stable isotopes values showed significant variation among species and detritus ratio, as well as their interaction; other effects were not significant (Table 1). For the detritus by ratio interaction, SCC’s were large and negative for δ^15^N and large and positive for δ^13^C suggesting both were important for multivariate effects. Within species, adults were generally more enriched in δ^15^N when grown either on animal detritus alone (2:0) or a 1:1 ratio of animal and plant detritus compared to the 2:10 ratio or plant only environments (Fig 4). *Aedes* were generally more enriched in δ^15^N compared to *Culex*. Specifically, *Aedes* grown in animal-only or plant only environments had higher values compared to *Culex*, with no differences among species in the 1:1 and 2:10 ratio (Fig 4). For δ^13^C, values for adults were highest in the 2:0 and 1:1 ratios, intermediate in the 2:10 ratio, and lowest in the 0:10 ratio for all species (Fig 4). The lowest values for δ^13^C and δ^15^N were found in the leaf-only environments for all species. Values of δ^15^N for pure crickets were generally higher compared to all species and detritus combinations except when larvae were grown in animal detritus alone (Fig 4).

**Table 1:** Results of multiple analysis of variance on δ^15^N and δ^13^C values in male and female *Aedes albopictus*, *A*. *aegypti*, and *Culex quinquefasciatus* across different ratios of plant and animal detritus. Significant effects are presented in bold.

				Standardized Canonical Coefficient	
Source	df	Pillai’s Trace	p-value	δ^15^N	δ^13^C
Species	4, 84	0.542	**< 0.001**	- 4.744	6.495
Detritus ratio	6, 84	1.9004	**< 0.001**	6.689	2.777
Sex	2, 41	0.052	0.334	7.412	1.866
Species x Ratio	12, 84	0.465	**0.024**	- 3.595	5.985
Species x Sex	4, 84	0.567	0.567	6.766	2.692
Ratio x Sex	6, 84	0.142	0.385	8.155	- 2.588
Species x Ratio x Sex	12, 84	0.218	0.592	- 5.625	5.283

**Fig 5:**
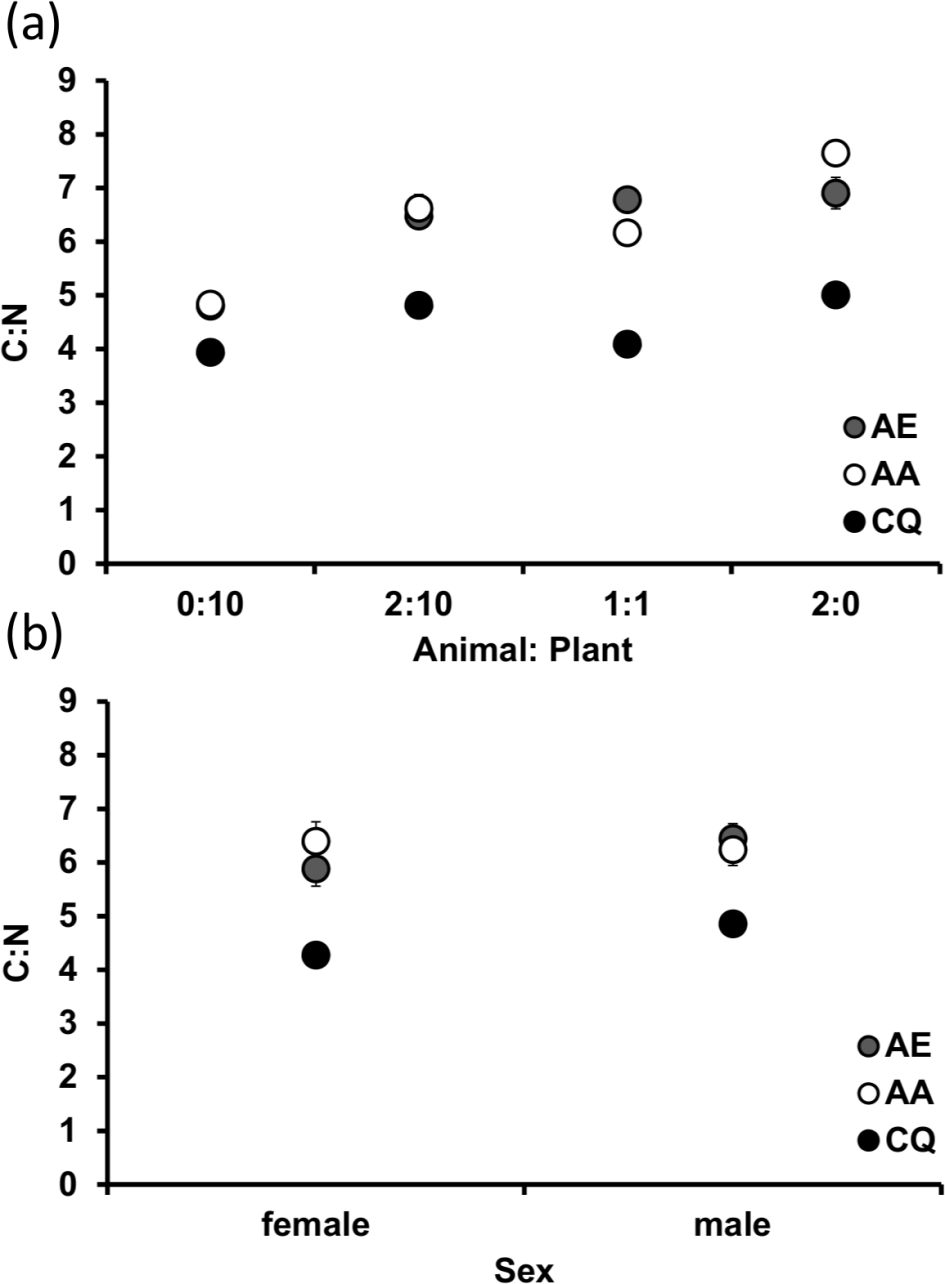
Ratio of tissue nitrogen (N) and carbon (C) for adult mosquitoes across different a) species and ratios (animal:plant) and b) species and sex. Values are means ± SE from three replicates (except AE 1:1 and CX 0:10 which each had only one sample). Detritus ratios are expressed in units, where one unit = 0.10 g.

As the statistical outcomes of the separate tests on nutrient values and the ratio of carbon to nitrogen were the same, we present the significant results for only the nutrient ratio (Table 2), and present the means for all species by sex by detritus combinations for both percent nitrogen and percent carbon (Table 3). These latter results were included to facilitate comparison with previous studies [8, 9]. The amount of carbon was similar between detritus types, however animal detritus contained more than three times the amount of nitrogen compared to plant detritus (Table 3). Results of MANOVA on carbon and nitrogen signatures in adult mosquitoes resulted in significant effects of species, sex, and detritus ratio, as well as interactions between species and sex and species and ratio (Table 2). For all effects, SCCs were generally larger for nitrogen compared to carbon. The results for the ratio of carbon to nitrogen (Table 2) paralleled the individual nutrient results (Table 3). Specifically, we detected a species (F_2,42_ = 137.35, P < 0.001), sex (F_1,42_ = 9.62, P = 0.003), and detritus ratio effect (F_3,42_ = 61.98, P < 0.001), as well as interaction between species and ratio (F_6,42_ = 4.18, P = 0.022) and species and sex (F_2,42_ = 4.99, P = 0.011) (Fig 5). For the species by ratio interaction, *A*. *aegypti* had a higher ratio in treatment levels containing animal detritus (2:10, 1:1, 2:0) compared to leaf alone (Fig 5A). *Aedes albopictus* showed a significant increase in their C:N ratio from 0:10 to 2:10 to 1:1 to 2:0 (Fig 5A). *Culex quinquefasciatus* showed less variation in the ratio across detritus treatments, with higher values in treatment levels with high levels of animal detritus (2:0, 2:10) compared to those with low (1:1) or no (0:10) animal detritus (Fig 5A). Differences were also evident among species within detritus levels. *Aedes* always had a higher ratio compared to *Culex* for all treatment levels (Fig 5A). There were no differences in ratios in carbon and nitrogen between male and female *Aedes aegypti* and *A*. *albopictus*, however all *Aedes* were different than *Culex* males and females, with male *Culex quinquefasciatus* also being significantly higher than female *C*. *quinquefasciatus* (Fig 5B).

**Table 2:** Results of multiple analysis of variance on nitrogen (mg) and carbon (mg) values in male and female *Aedes albopictus*, *A*. *aegypti*, and *Culex quinquefasciatus* across different ratios of plant and animal detritus. Significant effects are presented in bold.

				Standardized Canonical Coefficient	
Source	df	Pillai’s Trace	p-value	nitrogen	carbon
Species	4, 84	0.894	**< 0.001**	2.779	- 0.975
Detritus ratio	6, 84	0.822	**< 0.001**	2.585	- 1.205
Sex	2, 41	0.277	**0.001**	2.820	- 0.633
Species x Ratio	12, 84	0.424	**0.048**	2.814	- 0.880
Species x Sex	4, 84	0.251	**0.022**	- 1.777	1.471
Ratio x Sex	6, 84	0.037	0.953	1.150	0.876
Species x Ratio x Sex	12, 84	0.302	0.268	2.676	- 0.283

**Table 3:** Means (±SE) of percent carbon and nitrogen for male and female *Aedes aegypti*, *A*. *albopictus*, and *Culex quinquefasciatus* grown under different animal and leaf detritus ratios (1 unit of detritus = 0.05 g). Pure samples of plant and animal detritus are also included. Only one replicate for female *A*. *aegypti* in the 1:1 ratio was useable and only one replicate of *C*. *quinquefasciatus* in the 0:10 ratio produced enough adults to analyze. All other combinations are the result of three replicates.

Species	Sex	Animal: Plant	% carbon	% nitrogen
*Aedes aegypti*	male	2:0	55.45 ± 0.41	7.60 ± 0.26
	female		54.58 ± 1.61	8.48 ± 0.30
	male	1:1	52.11 ± 0.30	7.65 ± 0.17
	female		51.57	7.81
	male	2:10	53.27 ± 0.45	8.19 ± 0.02
	female		50.94 ± 0.07	7.94 ± 0.23
	male	0:10	47.21 ± 0.40	9.26 ± 0.08
	female		45.75 ± 0.84	10.19 ± 0.10
*Aedes albopictus*	male	2:0	54.81 ± 0.99	7.44 ± 0.06
	female		55.18 ± 0.79	6.98 ± 0.11
	male	1:1	52.17 ± 0.41	8.23 ± 0.09
	female		52.98 ± 1.46	8.85 ± 0.06
	male	2:10	50.30 ± 4.72	7.79 ± 0.17
	female		55.59 ± 1.12	8.19 ± 0.17
	male	0:10	43.59 ± 5.05	9.07 ± 0.62
	female		51.54 ± 3.87	10.63 ± 0.16
*Culex quinquefasciatus*	male	2:0	51.36 ± 1.02	9.63 ± 0.24
	female		49.51 ± 0.94	10.61 ± 0.12
	male	1:1	48.84 ± 0.63	11.48 ± 0.12
	female		49.96 ± 0.48	11.99 ± 0.16
	male	2:10	49.76 ± 0.64	9.51 ± 0.09
	female		50.25 ± 1.59	11.44 ± 0.09
	male	0:10	46.49	11.44
	female		44.08	11.56
Animal detritus			41.77 ± 5.38	7.99 ± 0.84
Leaf detritus			39.44 ± 2.96	1.25 ± 0.08

## Discussion

Like past studies on mosquito growth under different resource environments, we found that detrital environments composed of high quality animal, low quality leaf, or combinations of these types produced variation in male and female development times, mass, and survival among species [9, 33, 10]. Unlike past work, our study is the first to detail the underlying patterns of change in nutrient stoichiometry for multiple species and both sexes across these detrital environments. From this, we found support for our hypothesis that variation in animal and leaf detritus would alter the performance of mosquitoes, with generally high quality animal environments leading to faster development times, larger size, and higher survival compared to low quality leaf-only environments. *Culex quinquefasciatus* larval growth was similar to both *Aedes* in animal-only environments, however it performed worse, in terms of development times and survival, when leaf material was a part of, or the sole detrital source (Figs 1 and 3). Such differences in performance have been shown for *A*. *albopictus* and a different *Culex* species [9]. Beyond comparisons of plant and animal detritus, even variation in plant material quality has been shown to affect performance of *C*. *pipiens* and *A*. *albopictus* [31], suggesting that nutrient content alone may be more important than detritus type.

Nutrient stoichiometry was different between genera, and was manifest most in C:N ratios across detritus types (Fig 5A and Table 3). Specifically, *Aedes* tissues varied with changes in the C:N ratio and animal content of the detritus inputs. However, there was no change in the C:N ratio for *C*. *quinquefasciatus* adult tissue across treatments. Specifically, *C*. *quinquefasciatus* varied between 3.92:1 to 4.98:1 in plant and animal-only treatment levels, respectively, a change of 21%. However, *A*. *albopictus* and *A*. *aegypti* had changes in C:N of 37% and 30%, respectively, indicating a greater degree of flexibility in nutrient assimilation or allocation of assimilated resources. Another study identified similar differences for *A*. *albopictus* and *C*. *restuans*, with differences in C:N between leaf-only and animal-only environments of 47% and 17%, respectively [9]. Clearly this level of homeostasis for some *Culex* sits in stark contrast to the heterostatic patterns observed for the *Aedes* shown here. Although we did not statistically appraise differences, it appeared that both male and female *C*. *quinquefasciatus* grown in animal-only containers had shorter development times compared to either *Aedes* (Fig 1). This indicates that even though resources were high (which led to high survival and high mass of adults) the C:N ratio was lower for *C*. *quinquefasciatus* compared to *Aedes*. This may suggest that *C*. *quinquefasciatus* are less flexible in terms of development compared to *Aedes* or that they may sacrifice accumulating additional limiting nutrients in deference to faster emergence even when they are available. Another possibility is that *Aedes* concentrate more on accumulating lipid reserves, which would be reflective of their higher C:N ratios. Such difference in apparent flexibility in nutrient acquisition is intriguing, especially in light of interspecific resource competition, a subject that has been meticulously explored for container mosquitoes [31, 34, 45]. Future work, that considers the stoichiometric consequences of intra- and interspecific resource competition would be fruitful for our understanding of the determinants of mosquito production in nature.

Despite differences in detrital environments, we found less variation in stoichiometric patterns between *A*. *aegypti* and *A*. *albopictus*, than between both *Aedes* species and *C*. *quinquefasciatus* (Table 3 and Figs 4 and 5). The differences may point to several factors, including inherent differences in physiology or feeding behavior. Many studies have documented both intra- and interspecies differences in feeding behaviors among larvae of several *Culex* and *Aedes*, and how these differences may affect mosquito performance and production in containers [52–58]. Like most *Aedes*, larval *A*. *aegypti and A*. *albopictus* are predominantly browsers, spending more time near the middle or bottom of containers and using their mouthparts to remove (browse) microorganisms from surfaces. Alternatively *Culex* are filter feeders [53, 54] and species such as *C*. *quinquefasciatus*, and *C*. *coronator* [58], spend more time filtering the water column near the surface. As such, *Aedes* may more closely reflect the nutrient signature of detritus, whereas *Culex* appear to reflect water column microorganisms, which themselves subsist on detrital sources [9]. Our data on stable isotopes seems to support the conjecture that feeding behavior is an important explanation of nutrient profiles, as *Aedes*, and in most cases *A*. *albopictus*, were significantly more enriched in ^15^N compared to *C*. *quinquefasciatus* (Fig 4). *Anopheles*, which feed at the surface of the water, and *Culex quinquefasciatus*, were shown to exhibit resource partitioning based on stable isotopes of carbon and nitrogen, which the authors attributed to either feeding or assimilation differences [50]. However, feeding modes are not rigid and each taxon can switch between filtering and browsing depending on conditions [56, 58]. Differences in foraging behavior can affect mosquito performance under multiple detrital sources and their ability to obtain required and potentially limiting nutrients from these sources. For instance, *A*. *albopictus* has higher survival than *C*. *restuans* across many detritus ratios and was observed with lower tissue nitrogen concentration, probably due to greater foraging effort than *C*. *restuans* [9]. More work is needed to quantify how detrital processing affects mosquito larvae performance, but the ability to browse directly on detrital inputs does appear to benefit *Aedes* [59].

Nitrogen is often the limiting element in containers, an idea hypothesized based on the benefit to mosquito growth from additions of leaf-derived or soluble inputs of nitrogen in microcosm studies [60–64]. Moreover, others have found nitrogen to be essential for production of non-container mosquito species, where it can affect populations [65] and mosquito size [49]. In our study, detritus treatment levels varied in nitrogen availability, from the highest of 28.5 mg in the 2:10 level, to the lowest of 9.25 mg in the 1:1 level. Mosquito performance however did not respond to these values in a linear way. For instance, mass and development times for *C*. *quinquefasciatus* in the 1:1 ratio was similar to or exceeded the 0:10 ratio, even though the latter had more nitrogen (i.e., 12.5 mg). Moreover, the 2:0 ratio yielded the best performance for all species, even though it’s nitrogen content was not the highest (16.0 mg). Thus, total nitrogen input by itself does not seem to be predictive of mosquito development or mass, but how mosquito larvae acquire and assimilate that nitrogen as well as the nitrogen quality (which we did not measure) also may be more important. Additions of soluble nitrogen can affect mosquito performance through effects on fungal communities [63], suggesting that alternations in the microbial food sources of larvae may also play a role in their development. Phosphorous also may serve as a limiting resource [5], however it has been suggested that phosphorous may be important only in terms of its bioavailability [49]. The fact that ~50% of total body carbon and nitrogen of mosquitoes is structural and does not turnover within the lifetime of the mosquito [46] speaks to the importance of these elements in adult production.

Past work has shown considerable variation in trophic enrichment in container systems [8, 9]. Specifically, using a 0.5–1.0% fractionalization of ^13^C into the next higher trophic level, Winters and Yee [9] estimated ≥ 3 trophic levels between foraging mosquitoes and their leaf food source (+ 4.7 δ^13^C and + 3.9 δ^13^C for *A*. *albopictus* and *C*. *restuans*, respectively), and Kaufman et al. [8] using a similar approach, identified between 3–9 trophic levels for their work. Here we found identical values for *Aedes* compared to Winters and Yee [9] between leaf detritus and leaf reared *Aedes* (+ 4.7 δ^13^C) but + 5.0 δ^13^C for *C*. *quinquefasciatus*. In addition, using an approach of 2–3‰ enrichment of nitrogen to higher trophic levels, Hood-Nowotny et al. [49] found that direct grazing on detritus rather than microbial processing explained mosquito nitrogen composition. Although direct grazing on detritus alone is beneficial for container species, some species do appear to benefit from combined microbial and detrital contributions [59]. Other work in containers has indicated a strong link between microorganism communities and mosquito production [64]. Controversy still exists over the veracity of mean enrichment across trophic levels [46], but system level differences in food type, microbial communities, mosquito foraging behavior, and inherent differences in mosquito physiology likely explain some of these differences.

In a past study, Winters and Yee [9] found that mosquitoes reared on plant detritus showed depletion in ^15^N. We found a similar level of depletion here (Fig 4), although we do not have an explanation for this apparent consistent result. We would predict that consumers would become enriched in ^15^N relative to their food by 3–4‰, however the depletion of a heavier isotope in the tissue of leaf consuming invertebrates compared with the food source has been shown before (e.g., [66, 67]). Depletion of ^15^N compared with the source detritus may reflect the partitioning of nutrients into lipid or reproductive tissue [66] or be a consequence of microbial processing of the detritus before mosquito assimilation. A common container species not studied here, *Aedes triseriatus*, did exhibit a typical ^15^N pattern [8]; so species-level differences remains a possible explanation.

We hypothesized that males would have nutrient signatures different than females owing to differences in developmental rates, potentially linked to protandry. Unlike *Aedes* where no sex differences were found, male and female *C*. *quinquefasciatus* did exhibit differences in C:N, with males showing consistently lower concentrations of nitrogen compared to females across detrital environments (Table 3). Specifically, differences in the ratio of C:N (Fig 5) was explained by lower carbon but higher nitrogen in females compared to males. Sex-based investigations of nutrient signatures are uncommon for mosquitoes, however Hood-Nowotny and co-authors [49] compared stoichiometry profiles for nitrogen and phosphorous for male and female *Anopheles arabiensis*. These mosquitoes are found at the surface of larger bodies of water and are not considered container species. Females had an almost double requirement for phosphorous compared to males. The sexes were also different in fatty acid composition, which may be related to certain physiological processes like reproduction [68], although it is unclear what specific role different fatty acids had between the sexes. It was determined that N:P ratios where lower for females in seven species of *Drosophila*, perhaps owing to phosphorous demands during egg production [69]. We did not monitor phosphorous levels in this study, but these sex differences in nutrients may also have an underlying reproductive explanation.

We do not know why such differences in stoichiometry exist between species, although it is intriguing that there could be such variation among co-occurring genera, especially as many are of medical and veterinary importance. We can speculate that perhaps *Culex* and *Aedes* may simply solve the problems of resource acquisition and assimilation in different ways. How common homeostasis is among mosquitoes remains unknown. However, strict homoeostasis has been found for a non-container species, *Anopheles arabiensis*, where both males and females exhibited stable C:N ratios across multiple laboratory diets [49]. These authors concluded that C:N is a sex and species-specific fixed parameter, at least for that species. In our work, *A*. *albopictus* and *A*. *aegypti* both exhibited variation in C:N ratios, from a low of 4.83:1 in leaf-only containers to a high of 7.63:1 in animal-only containers for *A*. *albopictus*, a result that would argue against consistency in C:N ratios among mosquitoes. The implications of this work are more than differences among species or sexes we have identified here. Nutritionally stressed adults are generally smaller, take longer to develop, and exhibit a shorter life span [18, 70]. These small stressed adults also may be more likely to transmit virus [71], and can therefore be of greater medical importance. At present we have no data linking nutrient stoichiometry, adult life history parameters and growth, and viral competence. However, the ability to link nutrients signatures and propensity of those adults to spread disease is an unexplored but worthwhile area of research.

## References

[1] Sterner RW, Elser JJ (2002) Ecological Stoichiometry The Biology of Elements From Molecules to the Biosphere. Princeton University Press, New Jersey.

[2] Moore JC, Berlow EL, Coleman DC, Ruiter PC, Dong Q, Hasting A, et al (2004) Detritus, trophic dynamics and biodiversity. Ecol Lett 7: 584–600.

[3] Lounibos LP, Nishimura N, Escher RL (1993) Fitness of a treehole mosquito: influences of food type and predation. Oikos 66: 114–118.

[4] Kitching RL (2001) Food webs in phytotelmata: ‘bottom-up’ and ‘top-down’ explanations for community structure. Ann Rev Entomol 46: 729–760. 11112185 10.1146/annurev.ento.46.1.729

[5] Yee DA, Juliano SA (2006) Consequences of detritus type in an aquatic microsystem: assessing water quality, microorganisms, and the performance of the dominant consumer. Fresh Biol 51: 448–459. 10.1111/j.1365-2427.2005.01504.x PMC1862643 17476312

[6] Fish D, Carpenter SR (1982) Leaf litter and larval mosquito dynamics in tree-hole ecosystems. Ecology 63: 283–288.

[7] Walker ED, Olds EJ, Merritt RW (1988) Gut content analysis of mosquito larvae (Diptera: Culicidae) using DAPI stain and epifluorescence microscopy. J Med Entmol 25: 551–54. 10.1093/jmedent/25.6.551 2905011

[8] Kaufman MG, Pelz-Stelinski K, Yee DA, Juliano SA, Ostrom P, Walker ED (2010) Stable isotope analysis reveals detrital resource base sources of the tree hole mosquito, *Ochlerotatus triseriatus* . Ecol Entomol 35: 586–593. 21132121 10.1111/j.1365-2311.2010.01217.x PMC2995505

[9] Winters AE, Yee DA (2012) Variation in performance of two co-occurring mosquito species across diverse resource environments: insights from nutrient and stable isotope analyses. Ecol Entomol 37: 56–64.

[10] Murrell EG, Juliano SA (2008) The role of detritus type in interspecific competition and population distributions of *Aedes aegypti* and *Aedes albopictus* (Diptera: Culicidae). J Med Entmol 45: 375–383. 10.1603/0022-2585(2008)45[375:dtatoo]2.0.co;2 PMC2583230 18533429

[11] Kitching RL (2000) Food webs and container habitats The natural history and ecology of phytotelmata. Cambridge University Press, England.

[12] Yee DA (2008) Tires as habitats for mosquitoes: a review of studies within the eastern United States. J Med Entmol 45: 581–593. 10.1603/0022-2585(2008)45[581:tahfma]2.0.co;2 18714856

[13] Lounibos LP (2002) Invasions by insect vectors of human disease. Ann Rev Entomol 47: 233–266. 11729075 10.1146/annurev.ento.47.091201.145206

[14] Juliano SA, Lounibos LP (2005) Ecology of invasive mosquitoes: effects on resident species and on human health. Ecol Lett 8: 558–574. 17637849 10.1111/j.1461-0248.2005.00755 PMC1920178

[15] Mousson L, Dauga C, Garrigues T, Schaffner F, Vazeille M, Fallioux AB (2005) Phylogeography of *Aedes* (*Stegomyia*) *aegypti* (L.) and *Aedes* (*Stegomyia*) *albopictus* (Skuse) (Diptera: Culicidae) based on mitochondrial DNA variations. Gene Res 86: 1–11. 10.1017/S0016672305007627 16181519

[16] O’Meara GF, Evans LF Jr, Gettman AD, Cuda JP (1995) Spread of *Aedes albopictus* and decline of *Ae*. *aegypti* (Diptera: Culicidae) in Florida. J Med Entmol 32: 554–562. 10.1093/jmedent/32.4.554 7650719

[17] Braks MAH, Honório NA, Lounibos LP, Oliveira RL, Juliano SA (2004) Interspecific competition between two invasive species of container mosquitoes in Brazil. A Entomol Soc Am, 97: 130–139.

[18] Hawley WA, Reiter P, Copeland RS, Pumpuni CB, Craig GB Jr. (1987) *Aedes albopictus* in North America: probable introduction in used tires from northern Asia. Science 236: 1114–1116. 3576225 10.1126/science.3576225

[19] Mitchell CJ, Niebylski ML, Smith GC, Karabatsos N, Martin D, Mutebi JP, et al (1992) Isolation of eastern equine encephalitis virus from *Aedes albopictus* in Florida. Science 257: 526–527. 1321985 10.1126/science.1321985

[20] Ibaneze-Bernal S, Briseno B, Mutebi JP, Argot E, Rodriguez G, Martinez-Campos C, et al (1997) First record in America of *Aedes albopictus* naturally infected with dengue virus during the 1995 outbreak at Reynosa, Mexico. Med Vet Entomol 11: 305–309. 9430106 10.1111/j.1365-2915.1997.tb00413.x

[21] Gerhardt RR, Gottfried KL, Apperson CS, Davis BS, Erwin PC, Smith AB, et al (2001) First isolation of La Crosse virus from naturally infected *Aedes albopictus* . Emerg Infec Dis 7: 807–811. 11747692 10.3201/eid0705.017506 PMC2631884

[22] Turell MJ, O’Guinn ML, Dohm DJ, Jones JW (2001) Vector competence of North American mosquitoes (Diptera: Culicidae) for West Nile Virus. J Med Entmol 38: 130–134. 10.1603/0022-2585-38.2.130 11296813

[23] Turell MJ, Dohm DJ, Sardelis MR, O’Guinn ML, Andreadis TG, Blow JA (2005) An update on the potential of north American mosquitoes (Diptera: Culicidae) to transmit West Nile Virus. J Med Entmol 42: 57–62. 10.1093/jmedent/42.1.57 15691009

[24] Sprenger D, Wuithiranyagool T (1986) The discovery and distribution of *Aedes albopictus* in Harris County, Texas. J Am Mosq Cont Assoc 2: 217–219. 3507493

[25] Hobbs JH, Hughes EA, Eichold BH II (1991) Replacement of *Aedes aegypti* by *Aedes albopictus* in Mobile, Alabama. J Am Mosq Cont Assoc 7: 488–489. 1791461

[26] Livdahl TP, Willey MS (1991) Prospects for an invasion: competition between *Aedes albopictus* and native *Aedes triseriatus* . Science 253: 189–191. 1853204 10.1126/science.1853204

[27] Hornby JA, Moore DE, Miller TW Jr. (1994) *Aedes albopictus* distribution, abundance, and colonization in Lee County, Florida and its effect on *Aedes aegypti* . J Am Mosq Cont Assoc 10: 397–402. 7807083

[28] Barrera R (1996) Competition and resistance to starvation in larvae of container-inhabiting *Aedes* mosquitoes. Ecol Entomol 21:117–127.

[29] Teng HJ, Apperson CS (2000) Development and survival of immature *Aedes albopictus* and *Aedes triseriatus* (Diptera: Culicidae) in the laboratory: effects of density, food, and competition on response to temperature. J Med Entmol 37: 40–52. 10.1603/0022-2585-37.1.40 15218906

[30] Juliano SA, Lounibos LP, O’Meara GF (2004) A field test for competitive effects of *Aedes albopictus* on *Aedes aegypti* in south Florida: differences between sites of coexistence and exclusion? Oecologia 139: 583–593. 15024640 10.1007/s00442-004-1532-4 PMC1906877

[31] Costanzo KS, Mormann K, Juliano SA (2005) Asymmetrical competition and patterns of abundance of *Aedes albopictus* and *Culex pipiens* (Diptera: Culicidae). J Med Entmol 42: 559–570. 10.1093/jmedent/42.4.559 PMC1995070 16119544

[32] Allgood DW, Yee DA (2014) Influence of resource levels, organic compounds, and laboratory colonization on interspecific competition between *Aedes albopictus* and *Culex quinquefasciatus* (Diptera: Culicidae). Med Vet Entomol 28: 273–286. 10.1111/mve.12047 24444185 PMC4105337

[33] Daugherty MP, Alto BW, Juliano SA (2000) Invertebrate carcasses as a resource for competing *Aedes albopictus* and *Aedes aegypti* (Diptera: Culicidae). J Med Entmol 37: 364–372. 10.1093/jmedent/37.3.364 PMC2579927 15535579

[34] Juliano SA (2010) Coexistence, exclusion, or neutrality? A meta-analysis of competition between *Aedes albopictus* and resident mosquitoes. Israel J Ecol Evol 56: 325–351. 10.1560/IJEE.55.3-4.325 PMC3589801 23482823

[35] Costanzo K, Muturi E, Alto BW (2011) Trait-mediated effects of predation across life-history stages in container mosquitoes. Ecol Entomol 36: 605–615.

[36] Lopes J, Martins EAC, de Oliveira O, de Oliveira V, de Oliveira Neto BP, de Oliveira JE (2004) Dispersion of *Aedes aegypti* (Linnaeus, 1762) and *Aedes albopictus* (Skuse, 1894) in the rural zone of north Paraná State. Brazil Arch Biol Tech 47: 739–746.

[37] Yee DA, Allgood D, Kneitel JA, Kuehn KA (2012) Constitutive differences between natural and artificial container mosquito habitats: microorganisms, resources, and habitat parameters. J Med Entmol 49: 482–491. 10.1603/me11227 22679854

[38] Subra R (1981) Biology and control of *Culex pipiens quinquefasciatus* Say, 1823 (Diptera: Culicidae) with special reference to Africa. In Sci Appl 1: 319–338.

[39] Vinogradova EB (2000) *Culex pipiens pipiens* mosquitoes: taxonomy, distribution, ecology, physiology, genetics, applied importance and control Pensoft Pubishers, Sofia, Bulgaria.

[40] Foster WA, Walker ED (2002) Mosquitoes (Culicidae), pp. 245–249. In Mullen G, Durden L (editors). Med Vet Entomol. Academic Press New York, NY

[41] Yee DA, Skiff J (2014) Interspecific competition of a new invasive mosquito, *Culex coronator*, and two container mosquitoes, *Aedes albopictus* and *Cx*. *quinquefasciatus*, across different detritus environments J Med Entmol 51: 89–96. 10.1603/me13182 PMC3955010 24605457

[42] Kleckner CA, Hawley WA, Bradshaw WE, Holzapfel CM, Fisher IJ (1995) Protandry in *Aedes sierrensis*: the significance of temporal variation in female fecundity. Ecology 76: 1242–1250.

[43] Yee DA, Vamosi SM, Juliano SA (2012) Seasonal photoperiods alter developmental time and mass of an invasive mosquito, *Aedes albopictus* (Diptera: Culicidae), across its north-south range in the United States. J Med Entmol 49: 825–832. 10.1603/me11132 22897042

[44] Juliano SA (1998) Species introduction and replacement among mosquitoes: interspecific resource competition or apparent competition? Ecology 79: 255–268.

[45] Yee DA, Kaufman MG, Juliano SA (2007) The significance of ratios of detritus types and microorganism productivity to competitive interactions between aquatic insect detritivores. J Anim Ecol 76: 1105–1115. 17922707 10.1111/j.1365-2656.2007.01297.x PMC2579931

[46] Hood-Nowotny R, Knols BGJ (2007) Stable isotope methods in biological and ecological studies of arthropods. Entomol Exp Appl 124: 3–16.

[47] Goedkoop W, Akerblom N, Demandt MH (2006) Trophic fractionation of carbon and nitrogen stable isotopes in *Chironomus riparius* reared on food of aquatic and terrestrial origin. Fresh Biol 51: 878–886.

[48] Fry B (2006) Stable Isotope Ecology. Springer Science+Business Media, LCC, New York. 308 pp.

[49] Hood-Nowotny R, Schwarzinger B, Schwarzinger C, Soliban S, Madakacherry O, Aigner M, et al (2012) An analysis of diet quality, how it controls fatty acid profiles, isotope signatures and stoichiometry in the malaria mosquito *Anopheles arabiensis* . PLoS One 7: 1–15. 10.1371/journal.pone.0045222 PMC3484992 23133509

[50] Gilbreadth TA III, Kweka EJ, Afrane YA, Githeko AK, Yan G (2013) Evaluating larval mosquito resource partitioning in western Kenya using stable isotopes of carbon and nitrogen. Para Vect 6: 353. 10.1186/1756-3305-6-353 PMC3866463 24330747

[51] Scheiner SM (2001) MANOVA. Multiple response variables and multi species interactions In Scheiner SM, Gurevitch J (eds), Design and Analysis of Ecological Experiments. Oxford University Press, Oxford: 99–133.

[52] Pucat AM (1965) The functional morphology of the mouthparts of some mosquito larvae. Quaest Entomol 1: 41–86.

[53] Dahl C, Widahl L, Nilsson C (1988) Functional analysis of the suspension feeding system in mosquitoes (Diptera: Culicidae) Ann Entomol Soc Am 81: 105–127.

[54] Merritt RW, Dadd RH, Walker ED (1992) Feeding behavior, natural food, and nutritional relationships of larval mosquitoes. Ann Rev Entomol 37: 349–374. 1347208 10.1146/annurev.en.37.010192.002025

[55] Yee DA, Kesavaraju B, Juliano SA (2004) Interspecific differences in feeding behavior and survival under food-limited conditions for larval *Aedes albopictus* and *Aedes aegypti* (Diptera: Culicidae). Ann Entomol Soc Am 97: 720–728. 23197877 10.1603/0013-8746(2004)097[0720:IDIFBA]2.0.CO;2 PMC3507448

[56] Yee DA, Kesavaraju B, Juliano SA (2004) Larval feeding behavior of three co-occurring species of container mosquitoes. J Vect Ecol 29: 315–322. PMC2582444 15707290

[57] O'Donnell DL, Armbruster PC (2007) Comparison of larval foraging behavior of *Aedes albopictus* and *Aedes japonicus* (Diptera: Culicidae). J Med Entmol 44: 984–989. 10.1603/0022-2585(2007)44[984:colfbo]2.0.co;2 18047196

[58] Skiff J, Yee DA (2014) Behavioral differences among four co-occurring species of container mosquito larvae: effects of depth and resource environments. J Med Entmol 51: 375–381. 10.1603/me13159 PMC4011075 24724286

[59] Yee DA, Kesavaraju B, Juliano SA (2007) Direct and indirect effects of animal detritus on growth, survival, and mass of the invasive container mosquito *Aedes albopictus* (Diptera:Culicidae). J Med Entmol 44: 580–58. 10.1603/0022-2585(2007)44[580:daieoa]2.0.co;2 PMC2040033 17695011

[60] Carpenter SR (1982) Stemflow chemistry: Effects on population dynamics of detritivorous mosquitoes in tree-hole ecosystems. Oecologia 53: 1–6. 28310595 10.1007/BF00377128

[61] Walker ED, Lawson DL, Merritt RW, Morgan WT, Klug MJ (1991) Nutrient dynamics, bacterial populations, and mosquito productivity in tree hole ecosystems and microcosms. Ecology 72: 1529–1546.

[62] Walker ED, Kaufman MG, Ayres MP, Reidel MH, Merritt RW (1997) Effects on variation in quality of leaf detritus on growth of the eastern tree-hole mosquito, *Aedes triseriatus* (Diptera: Culicidae). Can J Zool 75: 706–718.

[63] Kaufman MG, Walker ED (2006) Indirect effects of soluble nitrogen on growth of *Ochlerotatus triseriatus* larvae in container habitats. J Med Entmol 43: 677–688. 10.1603/0022-2585(2006)43[677:ieosno]2.0.co;2 16892624

[64] Kaufman MG, Goodfriend W, Kohler-Garrigan A, Walker ED, Klug MJ (2002) Soluble nutrient effects on microbial communities and mosquito production in *Ochlerotatus triseriatus* habitats. Aqu Micro Ecol 29: 73–88.

[65] Duguma D, Walton WE (2014) Effects of nutrients on mosquitoes and an emergent macrophyte, *Schoenoplectus maritimus*, for use in treatment wetlands. J Vec Ecol 39: 1–13. 10.1111/j.1948-7134.2014.12063.x 24820550

[66] Wehi PM, Hicks BJ (2010) Isotopic fractionation in a large herbivorous insect, the Auckland tree weta. J Insect Physiol 56: 1877–1882. 10.1016/j.jinsphys.2010.08.005 20709068

[67] Crawley KR, Hyndes GA, Vanderklift MA (2007) Variation among diets in discrimination of δ^13^C and δ^15^N in the amphipod *Allorchestes compressa* . J Exp Marine Biol Ecol 349: 370–377.

[68] Lee RF, Hagen W, Kattner G (2006) Lipid storage in marine zooplankton. Marine Ecol Prog Ser 307: 273–306.

[69] Markow TA, Raphael B, Dobberfuhl D, Breitmeyer CM, Elser JJ, Pfeiler E (1999) Elemental stoichiometry of *Drosophila* and their hosts. Funct Ecol 13: 78–84.

[70] Reiskind MH, Lounibos LP (2009) Effects of intraspecific larval competition on adult longevity in the mosquitoes *Aedes aegypti* and *Aedes albopictus* . Med Vet Entomol 23: 62–68. 10.1111/j.1365-2915.2008.00782.x 19239615 PMC2651082

[71] Alto BW, Lounibos LP, Higgs S, Juliano SA (2005) Larval competition differentially affects arbovirus infection in *Aedes* mosquitoes. Ecology 8: 3279–3288. 10.1890/05-0209 PMC2605070 19096729

