# Correction: How Diverse Detrital Environments Influence Nutrient Stoichiometry between Males and Females of the Co-Occurring Container Mosquitoes *Aedes albopictus*, *Ae*. *aegypti*, and *Culex quinquefasciatus*

**DOI:** 10.1371/journal.pone.0144867

**Published:** 2016-03-23

**Authors:** Donald A. Yee, Michael G. Kaufman, Nnaemeka F. Ezeakacha

Fig 2, “Mass (mean ± SE) for male and female (a) *Aedes aegypti*, (b)*Aedes albopictus* and (c) *Culex quinquefasciatus*, across detritus ratios (animal:leaf),” incorrectly displays as a duplicate of Fig 1. Please see the correct Fig 2 here.

**Fig 2:**
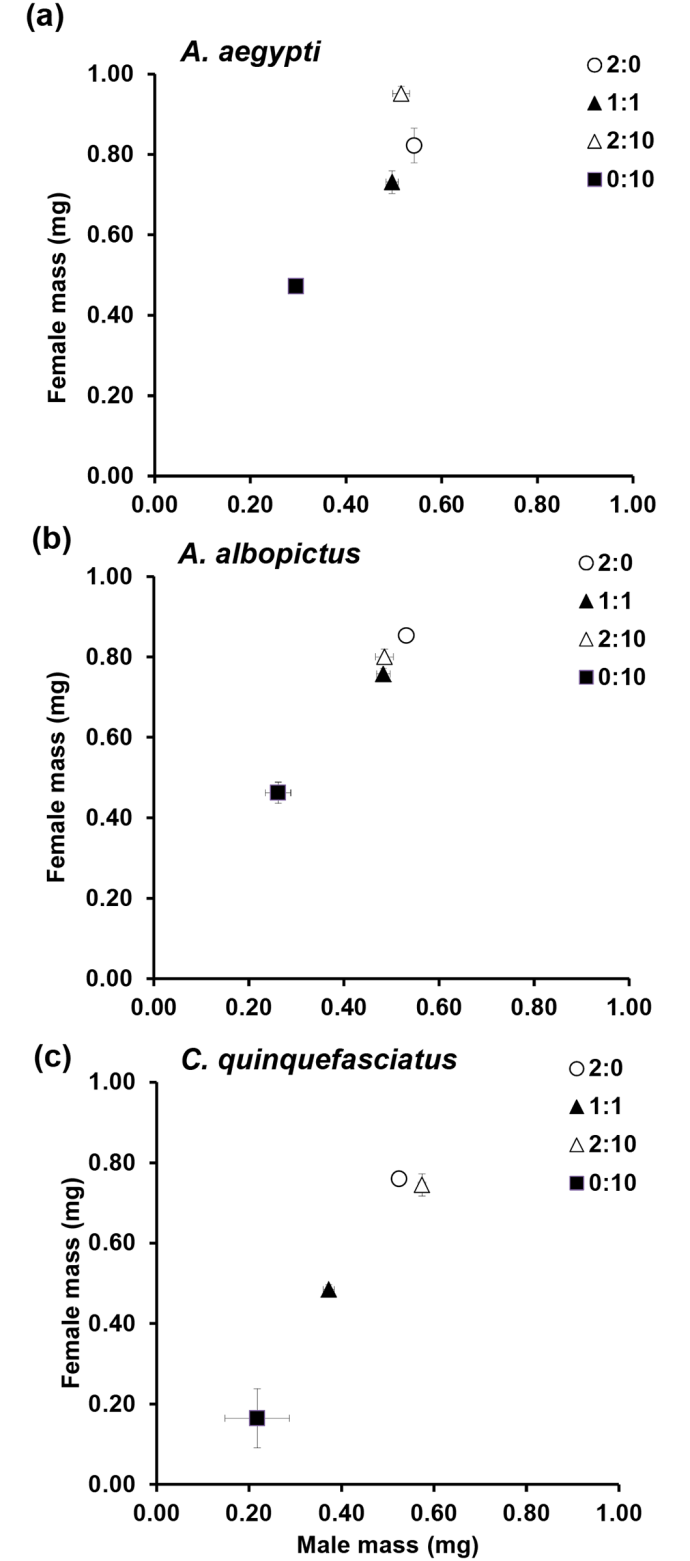
Mass (mean ± SE) for male and female (a) *Aedes aegypti*, (b)*Aedes albopictus* and (c) *Culex quinquefasciatus*, across detritus ratios (animal:leaf). Detritus ratios are expressed in units, where one unit = 0.10 g.
